# Isotype Diversification of IgG Antibodies to HIV Gag Proteins as a Therapeutic Vaccination Strategy for HIV Infection

**DOI:** 10.3390/vaccines1030328

**Published:** 2013-08-09

**Authors:** Martyn A. French, Laila N. Abudulai, Sonia Fernandez

**Affiliations:** 1 School of Pathology and Laboratory Medicine, University of Western Australia, Perth 6009, Australia; 2 Department of Clinical Immunology, Royal Perth Hospital and PathWest Laboratory Medicine, Perth 6000, Australia

**Keywords:** HIV, vaccine, HIV-1 Gag, IgG antibody diversification, IgG subclasses

## Abstract

The development of vaccines to treat and prevent human immunodeficiency virus (HIV) infection has been hampered by an incomplete understanding of “protective” immune responses against HIV. Natural control of HIV-1 infection is associated with T-cell responses against HIV-1 Gag proteins, particularly CD8^+^ T-cell responses restricted by “protective” HLA-B alleles, but other immune responses also contribute to immune control. These immune responses appear to include IgG antibodies to HIV-1 Gag proteins, interferon-α-dependant natural killer (NK) cell responses and plasmacytoid dendritic cell (pDC) responses. Here, it is proposed that isotype diversification of IgG antibodies against HIV-1 Gag proteins, to include IgG2, as well as IgG3 and IgG1 antibodies, will broaden the function of the antibody response and facilitate accessory cell responses against HIV-1 by NK cells and pDCs. We suggest that this should be investigated as a vaccination strategy for HIV-1 infection.

## 1. Introduction

The development of human immunodeficiency virus (HIV) vaccines is a global health priority, particularly at a time when therapeutic vaccines are being considered as a component of a strategy for eradicating HIV infection [1]. However, the development of therapeutic HIV vaccines has been hampered by an incomplete understanding of protective immune responses that control HIV infection, as exemplified by the failure of multiple candidate vaccines [2]. Here, we propose the hypothesis that isotype diversification of IgG antibodies against HIV-1 Gag proteins contributes to the control of HIV-1 replication and review the supporting evidence for this hypothesis, including data from our own studies. Furthermore, we discuss how this information might be applied to therapeutic vaccination strategies for HIV-1 infection.

## 2. Natural Control of HIV-1 Infection Is Associated with T-Cell Responses against HIV-1 Gag Proteins

Approximately 1% of patients with HIV-1 infection control the infection without antiretroviral therapy (ART) and are referred to as controllers [3]. Intense analysis of controllers is being undertaken to define “protective” immune responses against HIV-1 proteins that might be enhanced by therapeutic vaccines. Studies of HIV-1 controllers suggest that natural immune control of HIV-1 correlates with T-cell responses against viral proteins, particularly CD8^+^ T-cell responses against proteins of the virus core encoded by *Gag* that are restricted by “protective” HLA-B alleles [4,5], “helped” by Th1 CD4^+^ T-cells [6] and are “highly functional” [7]. Similarly, natural control of HIV-2 infection is also associated with high-magnitude polyfunctional Gag-specific CD8^+^ T-cell responses [8]. Of note, resting CD4^+^ T-cells “latently” infected with HIV-1 express Gag proteins on the cell surface more than other HIV proteins and are a potential target of immune responses against Gag proteins in patients receiving ART [9]. However, vaccine-induced CD8^+^ T-cell responses against HIV-1 Gag proteins have not been associated with prevention or control of HIV-1 infection in randomised controlled clinical trials involving large numbers of patients [2,10], though a clinical trial of an Ad5/Gag vaccine as a therapeutic vaccine did demonstrate that vaccine-induced Gag-specific CD4^+^ T-cells producing IFN-γ correlated with control of HIV-1 replication [11].

## 3. IgG Antibody Responses against HIV-1 Gag Proteins, Plasmacytoid Dendritic Cells and IFN-α-Dependant Natural Killer Cell Responses May Also Contribute to Control of HIV-1 Infection

Approximately one third of HIV-1 controllers do not exhibit evidence of HLA-B-restricted CD8^+^ T-cell responses against Gag proteins [12], suggesting that other immune responses also contribute to natural control of HIV-1 infection. At least 15 published studies undertaken between 1989 and 2000 in untreated HIV-1-infected adults and children who were not selected on the basis of a controller phenotype, demonstrated that progression of HIV-1 disease was slower in patients with higher serum levels and/or avidity of IgG antibodies to HIV-1 Gag proteins (p17, p24, p55) [13,14,15,16,17,18,19,20,21,22,23,24,25,26,27,28,29], suggesting that HIV-1 Gag proteins might be used as vaccine immunogens for eliciting antibodies to control HIV-1 infection. HIV-1 Gag proteins might have the particular advantage of exhibiting high intra-clade and inter-clade epitope conservation, at least for T-cell epitopes [30], and might thereby elicit broadly reactive antibodies. 

In addition, an increasing amount of evidence indicates that natural control of HIV-1 infection is associated with responses by interferon (IFN)-α-dependant natural killer (NK) cells [31] and plasmacytoid dendritic cells (pDCs) [32,33], which are the major producers of IFN-α [34]. Both NK cells and pDCs mediate innate immune responses against viruses [34,35], but both also function as accessory cells in IgG antibody responses, and therefore, their function might be enhanced by IgG antibodies induced by vaccines. Activation of both cell types induces a diverse anti-viral response that, in particular, includes lysis of virus-infected cells by NK cells and production of type I interferons by pDCs [34,35]. Plasmacytoid dendritic cells also function as antigen-presenting cells for T-cells [36,37,38,39], including cross-presentation to CD8^+^ T-cells [40,41], and regulate B-cell differentiation [42].

## 4. The Role of Non-Neutralising Antibodies in the Control of HIV-1 Infection

Non-neutralising antibodies mediate their effect by activating accessory cells, which also function as antigen-presenting cells and/or elicit innate immune responses. Activation of accessory cells by IgG non-neutralising antibodies is mediated by the Fc region of the antibody binding to Fcγ receptors [43]. Antibody responses of this type elicited against HIV-1 proteins include antibody-dependant NK cell responses (often referred to as antibody-dependant cell-mediated cytotoxicity; ADCC) [44,45], antibody-dependant cell-mediated viral inhibition (ADCVI) [46] and phagocytic antibodies [47,48]. It is currently unclear to what extent these antibody responses are associated with control of HIV-1 infection. Thus, whilst ADCVI responses to whole virus may be associated with prevention of HIV infection after vaccination with recombinant gp120 [46], they are not associated with prevention of HIV-1 superinfection [49]. Similarly, long-term slow progression of HIV-1 infection has been associated with a wide breadth of antibody-dependant NK cell responses to regulatory/accessory proteins of HIV-1 [50], but immune escape from ADCC antibodies to envelope proteins is common [45]. 

## 5. Diversification of IgG Antibody Responses against HIV-1 Gag Proteins May Broaden Fc Receptor Ligation and Accessory Cell Responses against HIV-1

Antibody-induced activation of NK cells (including ADCC) results from ligation of FcγRIIIa and is primarily mediated by monomeric or complexed antibodies of the IgG1 and IgG3 subclass, though complexed IgG2 and IgG4 antibodies can also bind to the 158V genotype of FcγRIIIa, which confers a higher affinity of Fc binding than the 158F genotype [51,52]. Plasmacytoid dendritic cells express the activatory receptor, FcγRIIa, as well as small amounts of the inhibitory receptor, FcγRIIb, in about 10% of healthy individuals, but not the activatory receptors, FcγRI or FcγRIIIa [53,54,55,56,57]. FcγRIIa plays a dominant role in phagocytic antibody responses [58] and has been demonstrated to facilitate the phagocytosis of immune complexes containing “self” or viral nucleic acids by pDCs, resulting in sensing of those nucleic acids by toll-like receptors and pDC activation [54,55,59].

Studies in patients with HIV-1 infection have demonstrated that FcγRIIa is the major FcR mediating phagocytosis of IgG antibodies complexed with gp120 [47]. FcγRIIa may be particularly effective in phagocytosis-induced activation of myeloid cells by immune complexes in HIV-1 infection, because, unlike other activatory FcγRs (FcγRI and FcγRIIIa), signal transduction via the immunoreceptor tyrosine-based activation motif (ITAM) of FcγRIIa does not require the FcR common γ-chain adaptor molecule, which is depleted by HIV-1 infection [60]. Support for this is provided by the observation that the 131H genotype of the FcγRIIa gene, which confers higher affinity Fc binding to FcγRIIa than the 131R genotype, is associated with slower progression of HIV-1 disease [61]. In contrast, the “high-affinity” 158V genotype of FcγRIIIa has been associated with an increased risk of acquiring HIV-1 infection [62,63] and also with an increased risk of HIV-1 disease progression [62], though the methods for analysing disease progression in that study are open to criticism. 

## 6. Diversification of IgG Antibodies against HIV-1 Gag Proteins to Include IgG2 Antibodies May Facilitate Ligation of FcγRIIa by Complexed Antibodies

Studies of immune complex binding to FcγRIIa *in vitro* demonstrate that all four subclasses of IgG are able to ligate the 131H genotype and, to a lesser extent, the 131R genotype of FcγRIIa, especially in the form of large immune complexes [51,52]. Although the affinity of ligation of FcγRIIa by IgG2 and IgG4 is less than that for IgG1 and IgG3, analyses of plasma immune complexes suggest that IgG2 antibodies play a particularly important role in the binding of immune complexes to FcγRIIa. IgG2 is the most abundant IgG isotype in plasma IgG/IgM immune complexes of healthy individuals and a disease-associated increase in the ratio of IgG3 to IgG2 in the immune complexes is associated with decreased binding to Fc receptors on myeloid cells [64]. We have shown that IgG2 is much more abundant than IgG1 in FcγRIIa-binding immune complexes from plasma of healthy individuals and HIV controllers, but that failure to control HIV-1 replication is associated with more abundant IgG1 in the immune complexes [48].

It is well-established that IgG2 antibodies and FcγRIIa play an important role in phagocytic antibody responses against polysaccharide antigens of encapsulated bacteria [65,66]. We suggest that IgG2 antibodies also contribute to phagocytic IgG antibody responses against antigens of persistent viruses, such as HIV-1, mediated via immune complexes and FcγRIIa expressed by pDCs. Targeting of viral antigens to FcγRIIa on BDCA-3^+^ dendritic cells by IgG antibodies has been proposed as a strategy for eliciting T-cell responses against viral antigens [67]. IgG2 is the only IgG subclass capable of covalent dimerization [68], which may enhance the function of this subclass of IgG antibody in phagocytosis and/or immune complex formation. In addition, IgG2 exhibits the highest degree of resistance to proteolytic degradation [69] and may also exhibit greater resistance than IgG1 to the adverse effects of deglycosylation of the Fc region on binding to FcγRIIa [70], though this was not confirmed in another study [52].

Support for our hypothesis that an IgG antibody response against HIV Gag proteins that has diversified to include IgG2 antibodies may be beneficial in the control of HIV-1 infection has been provided from studies in HIV-1 controllers or long-term non-progressors (LTNPs). Ngo-Giang-Huong *et al.* [71] examined plasma samples from 71 LTNPs, who had plasma HIV-1 RNA levels varying from <20 to 860,000 copies/mL and demonstrated that IgG2 antibodies to p55 and p24 were associated with lower plasma HIV-1 RNA levels. In contrast, plasma levels of IgG1 antibodies to these antigens did not correlate with HIV-1 RNA levels. We have examined plasma samples from 32 HIV-1 controllers, of whom 14 were elite controllers (plasma HIV RNA level <50 copies/mL), for IgG1 and IgG2 antibodies to HIV-1 proteins and shown that controllers had higher levels of IgG2 antibodies to Gag proteins than non-controllers and that this association was strongest in patients who did not carry the “protective” HLA-B57 allele [48]. In contrast, Banerjee *et al.* [72] examined serum from 16 HIV-1 controllers, of whom 13 had a plasma HIV-1 RNA level of <75 copies/mL, and demonstrated that although serum levels of total IgG and IgG1 antibodies to p24 were higher in HIV-1 controllers than patients with progressive HIV-1 disease, serum levels of IgG2 anti-p24 did not differ between HIV-1 controllers and patients with progressive HIV-1 disease. It is unclear why control of HIV-1 replication was associated with IgG2 antibodies against HIV-1 Gag proteins (p55 and/or p24) in two studies [48,71], but only with IgG1 antibodies to HIV-1 p24 in another [72]. Differences might reflect the use of Western blot assays and antigens from virus lysates in the studies by Ngo-Giang-Huong *et al.* [71] and French *et al.* [48], as opposed to ELISAs and recombinant HIV-1 proteins in the study by Banerjee *et al.* [72]. Furthermore, the study of HIV-1 controllers by Banerjee *et al.* [72] did not subgroup patients according to carriage of “protective” HLA-B alleles.

In summary, we suggest that diversification of an IgG antibody response against HIV-1 Gag proteins to include IgG2 antibodies, as well as IgG3 and IgG1 antibodies, may enhance the activation of accessory cell immune responses by NK cells and pDCs via ligation of both FcγRIIIa and FcγRIIa. 

## 7. Isotype Diversification of IgG Antibodies to Core or Capsid Proteins of Other Persistent Viruses Is Associated with Control of Infection

Further support for our hypothesis that isotype diversification of IgG antibodies against HIV-1 Gag proteins is associated with control of HIV-1 infection is provided by evidence from patients infected by other persistent viruses. Data from patients with acute hepatitis C virus (HCV) infection suggests that IgG2 antibodies to HCV core proteins might be associated with clearance of HCV infection. Zein *et al.* [73] reported that all of the four patients who spontaneously cleared HCV infection had IgG2 antibodies to HCV core proteins compared with only nine of 23 patients who did not clear the infection. Furthermore, the ratio of IgG2/IgG1 HCV core-specific antibody titres was >1 in three of the four patients. In addition, studies in patients with human papillomavirus (HPV) infection demonstrated that IgG2 antibodies to capsid proteins were associated with protection from HPV disease using an ELISA and virus-like particles as antigens [74], though IgG2 antibodies could not be detected at all in another study when capsid proteins were used as antigens [75]. 

## 8. Regulation of IgG Antibody Isotype Diversification and the Effect of HIV Infection

Isotype diversification of IgG antibody responses occurs during the process of B-cell differentiation and maturation of the antibody response, which occurs in germinal centres of lymphoid tissue follicles following the interaction of naive B-cells with follicular dendritic cells and follicular-helper T-cells (T_FH_-cells) [76]. Immunoglobulin isotype switching during B-cell differentiation occurs through class switch recombination of immunoglobulin heavy chain genes, with switching to IgG2 and IgG4 occurring “downstream” of IgG3 and IgG1 [77], and results in broadening of IgG antibody function mediated by the Fc region (Table 1). Together, IgG1 and IgG2 comprise about 90% of serum IgG [78] and, therefore, exert the largest functional effect on an IgG antibody response. 

**Figure 1:**
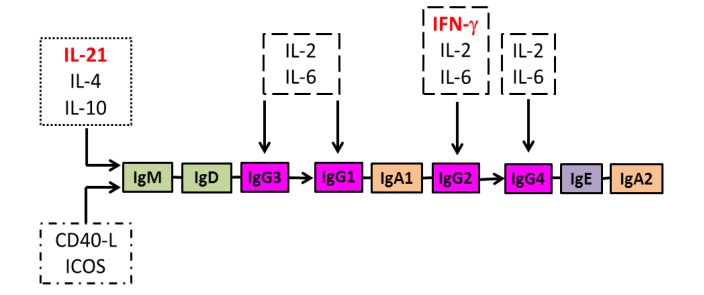
Isotype diversification of an IgG antibody response. IgG antibody isotype switching during B-cell differentiation in germinal centres results from class switch recombination of immunoglobulin heavy chain genes from “downstream” (IgG3 and IgG1) to “upstream” (IgG2 and IgG4) isotypes regulated by co-stimulatory molecules (CD40L and inducible co-stimulator (ICOS)) and cytokines (IL-4, IL-10 and IL-21). Pro-inflammatory cytokines (IL-2, IL-6 and IFN-γ) enhance immunoglobulin production with IFN-γ particularly increasing IgG2 production. CD4^+^ T-cell production of both IL-21 and IFN-γ is impaired by HIV infection.

Regulation of immunoglobulin isotype switching is mediated primarily by molecules expressed on, or produced by, T_FH_-cells [76]. The most important are the co-stimulatory molecules, CD40 ligand and inducible co-stimulator (ICOS), as exemplified by the association of immunoglobulin deficiency with deficiency of these molecules [79,80], and the cytokines, IL-4, IL-10 and IL-21, as exemplified by the restoration of immunoglobulin production by B-cells from patients with IgA deficiency or common variable immunodeficiency disorder when cultured with these cytokines [81,82,83]. The co-inhibitory molecule programmed death (PD)-1 is also highly expressed by T_FH_-cells, and ligation by the ligand PD-L1 has been shown to down-regulate ICOS expression and IL-21 production and possibly contribute to T_FH_-cell dysfunction caused by HIV infection [84]. Pro-inflammatory cytokines, such IL-2, IL-6 and IFN-γ, also contribute to isotype diversification of IgG antibodies, but primarily by enhancing production of IgG subclasses rather than initiating isotype switching [85,86,87,88,89] (Figure 1).

**Table 1:** Isotype diversification of IgG antibodies leads to broadening of the function of an IgG antibody response.

IgG3	IgG1	IgG2	IgG4
Ligation of all Fc receptors, including FcγRI in monomeric form (IgG3 > IgG1)		Restricted ligation of Fc receptors and only when complexed, particularly large complexes	Restricted ligation of Fc receptors and only when complexed, particularly large complexes
Potent complement activation through the classical pathway (IgG3 > IgG1)		Weak complement activation	No complement activation
		Most resistant of all IgG isotypes to proteolytic degradation	Produced after chronic immune stimulation, particularly parasite infections
		Predominant IgG subclass in plasma IgM-IgG complexes	Regulated similarly to IgE
		Only IgG subclass to undergo covalent dimerization	May form bispecific antibodies
		Predominant IgG subclass in phagocytic antibodies to polysaccharide antigens	

While B-cell activation and increased production of total IgG is characteristic of HIV infection, driven to a large degree by pro-inflammatory cytokines [90], IgG2 deficiency is common in HIV patients [47,91], and IgG2 and IgA are less abundant in lymph node germinal centres of HIV patients than controls [92]. Indeed, serum levels of the “upstream” isotypes, IgG3 and IgG1, are increased, whereas serum levels of the “downstream” isotypes, IgG2 and IgG4, are decreased in HIV patients [90,93], suggesting an acquired disorder of B-cell differentiation and isotype diversification similar to that in patients with primary antibody deficiency disorders [94]. 

Data from studies of cytokine regulation of IgG subclass production by B-cells [85,86,88] and of patients with IgG2 deficiency [89] indicate that IFN-γ plays a particularly important role in the production of IgG2. Decreased IgG2 production in HIV patients may therefore be a consequence of both impaired B-cell isotype switching associated with T_FH_-cell dysfunction [84,95] and impaired IFN-γ production that characterises HIV-induced immunodeficiency, but is preserved in HIV controllers [6]. We provided evidence in support of this proposal from a study of antibody responses to HIV p24 in ART-treated HIV patients enrolled into a clinical trial of a recombinant DNA vaccine encoding a fowlpox virus vector, HIV Gag-Pol and IFN-γ [96]. Although the number of patients was small, this study provided evidence that the vaccine construct containing the gene for IFN-γ increased IgG antibodies to HIV p24, including IgG2 antibodies, which were associated with better control of HIV replication after ART was ceased in patients who possessed the 131H genotype of FcγRIIa, which results in the highest affinity binding of IgG2 antibodies to that receptor. 

It is notable that lymph node T_FH_-cells of patients with HIV-1 infection exhibit greater reactivity with Gag proteins than Env proteins [97]. Dysfunction of T_FH_-cells associated with HIV-1 infection [84,95,97] may therefore contribute to limited isotype diversification of IgG antibodies against HIV-1 Gag proteins.

## 9. Potential Strategies for Enhancing Isotype Diversification of IgG Antibodies to HIV-1 Gag Proteins

Therapeutic modulation of the isotype of vaccine-induced IgG antibodies is not an established procedure in humans, but has been achieved in dogs with a saponin-adjuvanted *Leishmania* vaccine [98]. Preliminary data from patients with HIV-1 infection suggest that IFN-γ might enhance vaccine-induced IgG2 antibodies to HIV-1 Gag proteins [96], and this potential approach to therapeutic vaccination should be considered further. Finally, inhibition of immune activation in HIV-1 patients by PD-1 blockade might also have beneficial effects on T_FH_-cell function [84] and antibody responses [99], and examination of IgG antibody isotype diversification might be examined in clinical trials of therapies that block the PD-1/PD-L1 pathway. 

## 10. Conclusions

**Figure 2:**
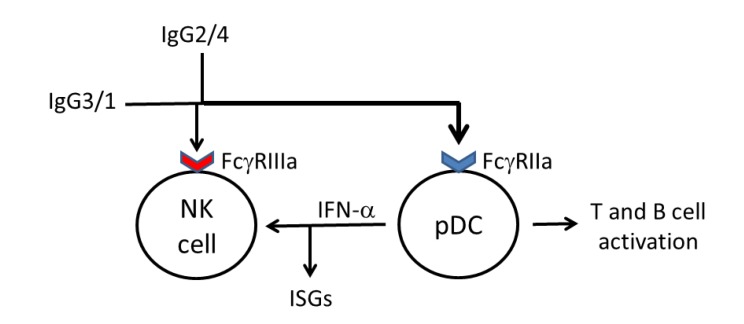
A diagrammatic representation of how isotype diversification of IgG antibodies against HIV-1 Gag proteins might enhance anti-viral accessory cell responses against HIV-1 infection. It is proposed that IgG antibodies bind to HIV-1 Gag proteins expressed on the surface of cells infected by HIV-1, including resting CD4^+^ T-cells [9]. Activation of natural killer (NK) cells is elicited by “downstream” IgG isotypes (IgG3 and IgG1) via FcγRIIIa. “Upstream” IgG isotypes (IgG2 and possibly IgG4) may also contribute to NK cell activation by ligating FcγRIIIa, particularly in individuals carrying the 158V genotype. However, it is proposed that multimeric IgG2 antibodies primarily broaden the function of the antibody response by enhancing phagocytic activity against Gag proteins associated with HIV-1 RNA, as a consequence of the functional characteristics of IgG2 (see Table 1), which activates plasmacytoid dendritic cells (pDCs) via FcγRIIa. Activation of pDCs leads to the production of IFN-α, which facilitates NK cell responses and induces the production of interferon-stimulated genes (ISGs) and to antigen presentation and/or stimulation of B- and T-cells (see text). HIV-1 infection impairs diversification of an IgG antibody response to “downstream” isotypes.

We propose that enhancing isotype diversification of IgG antibody responses against HIV-1 Gag proteins during vaccination, to include IgG2, as well as IgG3 and IgG1 antibodies, may result in an IgG antibody response that facilitates the accessory cell responses of NK cells and pDCs to elicit both ADCC responses by NK cells, as well as phagocytosis of complexed antibody by pDCs and a pDC-dependant antiviral response (Figure 2). Further experimental evidence is required to strengthen our hypothesis. In particular, studies are needed to establish that IgG2 antibodies inhibit HIV-1 replication and are not just a marker of Th1 responses. However, at a time when new approaches to the development of HIV vaccines are needed [2], we suggest that consideration should be given to vaccination strategies that will enhance isotype diversification of IgG antibodies against HIV-1 Gag proteins.
